# Gate Length Variation Effect on Performance of Gate-First Self-Aligned In_0.53_Ga_0.47_As MOSFET

**DOI:** 10.1371/journal.pone.0082731

**Published:** 2013-12-18

**Authors:** Mohd F. Mohd Razip Wee, Arash Dehzangi, Sylvain Bollaert, Nicolas Wichmann, Burhanuddin Y. Majlis

**Affiliations:** 1 Institute of Electronics, Microelectronics and Nanotechnology (IEMN), University Lille 1, Villeneuve d'Ascq, France; 2 Institute of Microengineering and Nanoelectronics (IMEN), Universiti Kebangsaan Malaysia, Bangi, Selangor, Malaysia; Gazi University, Turkey

## Abstract

A multi-gate n-type In_0.53_Ga_0.47_As MOSFET is fabricated using gate-first self-aligned method and air-bridge technology. The devices with different gate lengths were fabricated with the Al_2_O_3_ oxide layer with the thickness of 8 nm. In this letter, impact of gate length variation on device parameter such as threshold voltage, high and low voltage transconductance, subthreshold swing and *off* current are investigated at room temperature. Scaling the gate length revealed good enhancement in all investigated parameters but the negative shift in threshold voltage was observed for shorter gate lengths. The high drain current of 1.13 A/mm and maximum extrinsic transconductance of 678 mS/mm with the field effect mobility of 364 cm^2^/Vs are achieved for the gate length and width of 0.2 µm and 30µm, respectively. The source/drain overlap length for the device is approximately extracted about 51 nm with the leakage current in order of 10^−8^ A. The results of RF measurement for cut-off and maximum oscillation frequency for devices with different gate lengths are compared.

## Introduction

The continuous scaling of MOS devices leads to some fundamental limits such as short channel effects (SCEs) and high leakage current related to having lower gate controllability on the channel. This can make a crucial challenge against the performance improvements of the scaled devices mentioned by the International Technology Roadmap of Semiconductors (ITRS). To conquer this limitations, several new technologies such as high-k dielectrics [1], metal gate electrodes [2], stressors [3], and new transistor architectures based on silicon-on-insulator (SOI), such as Fin FETs [4], Junctionless transistors [5] or gate-all-around FETs [6], have been proposed. Another important option, in order to overcome the scaling limitation, is to seek any possible alternative of “beyond Si” channel materials, such as Germanium and III–V compound semiconductors. In this matter, ternary III–V compound InGaAs is considered as a reliable material for future CMOS devices, regarding to its high electron mobility, saturation velocity, achievable band gap engineering and narrow band gap in comparison with the Si or GaAs base device counterparts.

In fact, reduction of gate dimension requires decrease of the oxide thickness, which may lead to unwanted gate leakage current. In order to reduce gate current, high permittivity (high-*k*) dielectrics has been considered with the ability of being ultra-thin insulator beyond the SiO_2_ probable limitations. The proper high-k dielectric material must be thermally stable up to 1000 ° C since it is subjected to annealing at high temperature during the fabrication process of the transistor. Recently, the development of atomic-layer-deposited (ALD) technology has provided a promising result for depositing ultra-small thickness of the oxide layers. As a proper candidate for dielectrics on III-V semiconductors, several dielectrics has been recently proposed, such as ALD Al_2_O_3_ [7], [8], HfO_2_ [9] or HfAlO [10]. Some high performance devices have been reported for self-aligned InGaAs MOSFETs with high-k gate dielectrics formed by ALD [11], [12], [13].

A gate-first self-aligned process is required to reach high speed logic devices by reducing overlap capacitance and series resistance [14]. Lower series resistance can supress loss of the drain current by decreasing the gate and source/drain misalignment. Moreover, a gate-first method has less complication at fabrication process in comparison with the gate-last process. However, the gate-first process imposes more thermal budget over the device and introduces larger interface trap density between the high-*k* /InGaAs interface.

Due to the higher resilience against the drain induced-barrier-lowing effects or leakage problems, the inversion type MOSFETs are more preferred than depletion type MOSFETs with buried-channel [7]. In previous work [15], the fabrication of inversion type In_0.53_Ga_0.47_As MOSFET with 8 nm Al_2_O_3_ gate oxide thickness, using ALD, was briefly reported. It is found that the devices with Al_2_O_3_ oxide layer has less interface trap density (D_it_) compare to the ones with HfO_2_ [7]. Moreover, Al_2_O_3_ has a high band gap (*∼*9 eV), a high-breakdown electric field (5–30 MV/cm), and a satisfactory result in terms of equivalent oxide thickness (EOT) with high thermal stability (up to at least 1000°C) [8]. In most of the reported cases for self-aligned InGaAs MOSFETs, it was used refractory metals as the gate metal in fabrication process. The gate material used in this work is Tantalum (Ta) whose high resistivity value (1.8×10^−6^ Ωm) can interrupt extraction of accurate small signal equivalent circuit. To avoid this problem a multi-gate technology is implemented to define multi fingers for present work. In multi finger structure the gate resistance is decreased by the factor of 1/n^2^, where n in the number of fingers. The devices have 8 fingers with the air bridge to connect all the sources in coplanar topology.

In this work, the fabrication process of inversion mode n-type In0.53Ga0.47As MOSFET is elaborately addressed and the electrical characterization of the device is developed. The impact of length variation on threshold voltage, high and low drain voltage transconductance, output characteristics, gate leakage current and field effect mobility are demonstrated. Threshold voltages, subthreshold swing, *off* drain current and gate to source/drain overlap length are extracted and compared for all devices with different lengths down to 200 nm. Finally, the RF results for cut-off and maximum oscillation frequency of devices with different gate lengths are shown and compared.

## Methodology

**Figure 1:**
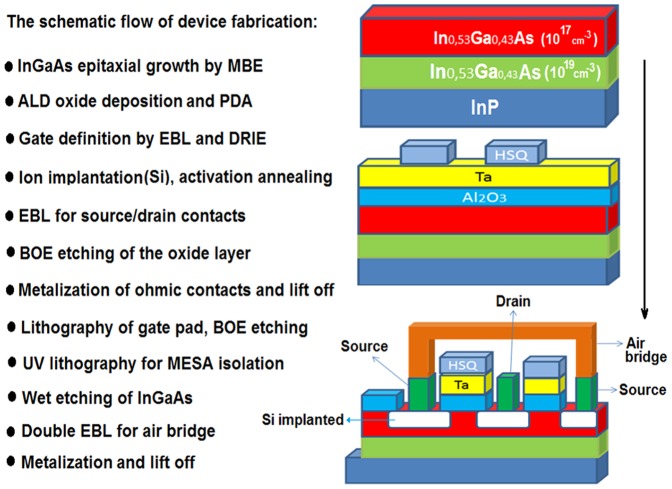
Schematic flow of fabrication for self-aligned n-type In_0.53_Ga _0.47_As with the air bridge.

The schematic flow of fabrication for self-aligned n-type In0.53Ga0.47As MOSFET is illustrated in Fig 1. The device’s fabrication began with a molecular beam epitaxy (MBE model RIBER 32P) growth of 2 layers of In_0.53_Ga_0.47_As on InP substrate. The top layer is C-doped with the concentration of 1×10^17^/cm^3^ and the thickness of 300 nm. The second layer as a buffer has thickness of 500 nm with C-doped concentration of 1×10^19^/cm^3^. Once completed, a wet passivation treatment using ammonia took place prior to the oxide deposition. Ammonia solution was diluted to obtain 5% of concentration and the wafer was dipped for 5 minutes. Then, 8 nm of Al_2_O_3_ was deposited by ALD (BENEQ-TFS200) technique.

During the process, substitution pulsing of the liquid precursor of Trimethylaluminium (TMA) and H_2_O were executed at 300°C. Argon purging was introduced between each pulse to remove the excess of materials. This was followed by post deposition annealing (PDA) at 600 °C with nitrogen flux. PDA at high temperature is recommended after the oxide deposition by ALD to activate the dopants and improve the interface between the oxide and InGaAs to minimize fixed costs of the flash annealing. To define the gate contacts, a metallic layer of Tantalum (Ta) was deposited with sputtering technique and the thickness was fixed at 200 nm. Argon was flown in the chamber to create a plasma condition. To achieve sub-micron dimension in our transistor, electron beam lithography (EBL) is needed for patterning the gate. Hidrogen silsesquioxane (HSQ) photoresist was deposited with a standard spin coater. Before HSQ deposition, hexamethyldisilazane (HDMS) was coated to improve the adhesion of HSQ on the substrate. Next step is e-beam exposure with appropriate dose for different gate lengths. The gate width of 30 µm was fixed for all gate lengths.

Deep Reactive Ion Etching (DRIE) technique was performed to remove the undesired Ta on the structure using Oxford Plasma Lab System 100. The most critical step in fabrication process is the ion implantation, where Silicon was doped in In_0.53_Ga_0.47_As layer with the energy of 15 KeV. The dose of implantation ( =  5×10^13^at/cm^2^) was chosen based on the calculation from a software called TRIM. During the source and drain doping, the gate can protect the channel from ion implantation. To finish with implantation, activation annealing at 750°C during one minute was performed, which was vital for dopants redistribution.

E-beam lithography process was repeated in order to define source –drain contacts before Al_2_O_3_ layer was etched using buffered oxide etch (BOE). The revelation of the structure was done using methyl isobutyl ketone (MIBK) and isopropyl alcohol (IPA). Later, a metallization of Ti/Pt/Au: 250/250/3000Å stack as the ohmic contacts were performed by evaporation, and accompanied by the post metallization annealing at 400°C using forming gas. To finish this step, the structure was lifted –off using acetone and alcohol.

**Figure 2:**
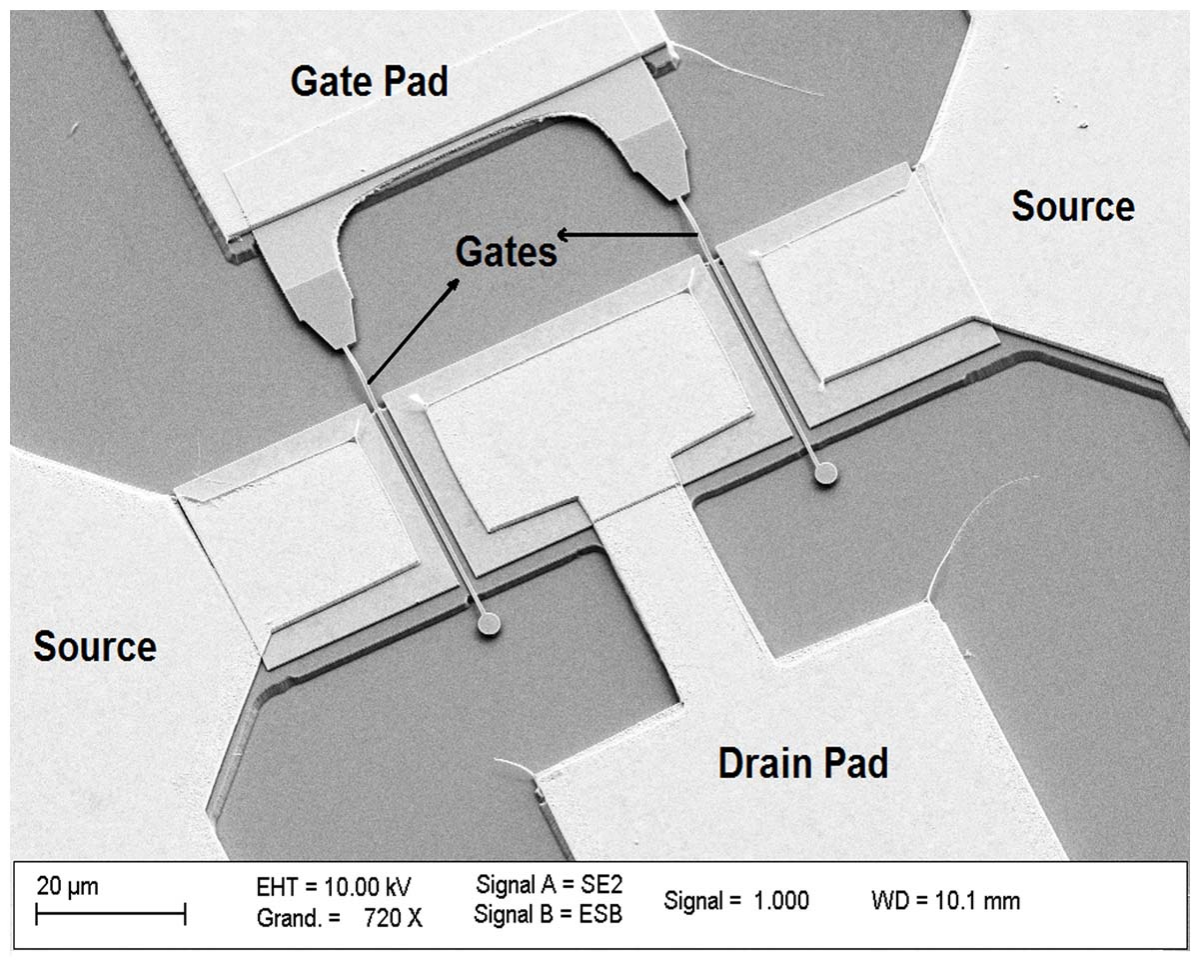
SEM image of In_0.53_Ga _0.47_As MOSFETs with two fingers.

Then, another optical lithography was required to realize the device’s gate pad which is essential for measurement. For MESA isolation, a basic UV lithography was first conducted before anisotropic wet etching of In_0.53_Ga_0.47_As layer for isolation of ohmic contacts. The solution used is H_3_PO_4_: H_2_O_2_: H_2_O with a ratio of 5:1:40. In Fig 2, the final structure of In_0.53_Ga_0.47_As MOSFET with two fingers is shown.

**Figure 3:**
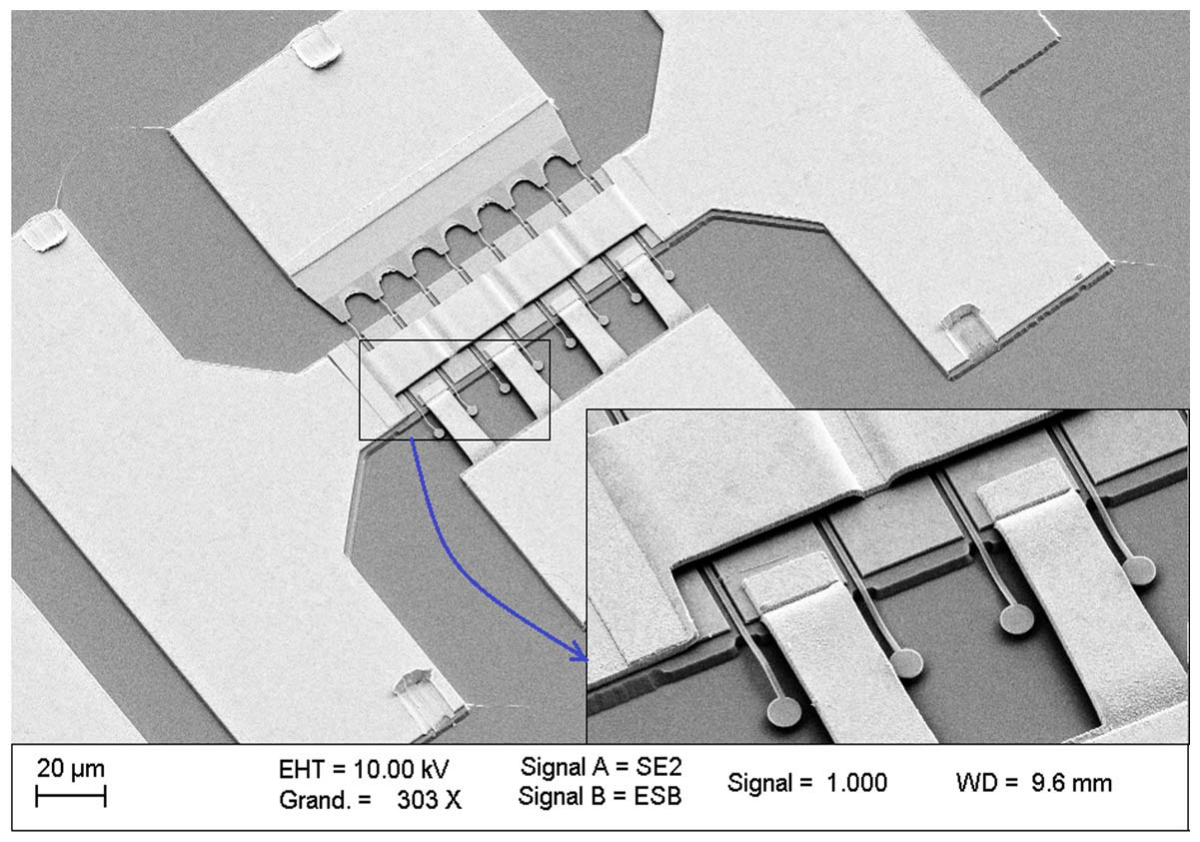
SEM image of In_0.53_Ga _0.47_As MOSFETs with air bridge and 8 fingers.

As it is mentioned before, multi-gate structure was used to avoid the impact of high resistivity value of Ta, as the gate material, on the gate resistance. In this matter, an air bridge technology using double E-beam lithography was realized to make a connection between source contacts. At the first lithography for the pier of the bridge, we used PMGI SF11 and the developer was NANO 101. The second lithography was similar to the previous one for source-drain definition, which used the combination of PMMA and copolymer MAA-MMA. Finally, the process was completed with a metallization of Ti/Au (1000/7000Å). Like previously mentioned, lift off was applied but with different solution which is Remover PG at temperature of 80°C followed by acetone and IPA. Fig. 3 demonstrates the n-type In_0.53_Ga_0.47_As MOSFET with the air bridge and 8 fingers. For electrical characterization of the devices, the Agilent Network Analyser (E5270B-67GHz) equipped with the infinity probe (CASCADE, Microtech.Inc) was implemented.

## Results and Discussion

All DC measurements were carried out at room temperature. The Transmission Line Measurement (TLM) can enable us to measure the resistance value and quality of the ohmic contacts. By TLM method at room temperature, the parameters for sheet resistance of implanted region and contact resistance were calculated as 112 Ω/square and 0.2 Ω.mm respectively.

**Figure 4:**
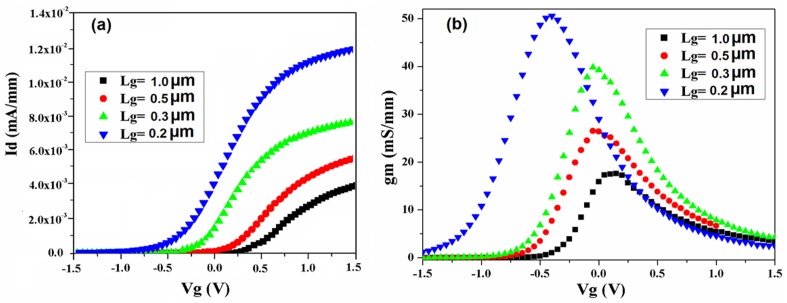
I_d_-V_g_ characteristics (a) and Transconductance vs gate voltage (b) for In_0.53_Ga _0.47_As MOSFETs with different L_g_ for low drain voltage (V_d_  =  50 mV, T = 300 K).

Typical transfer characteristics (I_d_-V_g_) curve leads to measuring of several device parameters, e.g. threshold voltage (V_th_ ), sub-threshold slope (SS) or *off*-state leakage current (*I_doff_*). These parameters reveal the device performance in accordance to the device scaling. The transfer characteristics and transconductance (g_m_, as a function of the gate voltage), of the self-aligned In_0.53_Ga _0.47_As devices with different gate lengths (L_g_) down to 0.2 µm, are shown in Fig 4a,b. Devices were biased in linear regime of operation with low drain voltage (V_d_ =  50 mV). The results reveal significant increasing in drain current (I_d_) by scaling the L_g_ down to 200 nm (Fig 4a). Moreover, g_m_ measurement (Fig 4b) shows the same trend but the peaks of the g_m_ shift to negative gate voltage by reduction of the L_g_. This behaviour can imply the negative shift in threshold voltage (V_th_) due to the scaling of the gate length, which is in agreement with the results shown in Id-Vg graph (Fig 4a). By linear extrapolation of the transfer characteristics V_th_ can be extracted which confirm the negative shift in V_th_.

**Figure 5:**
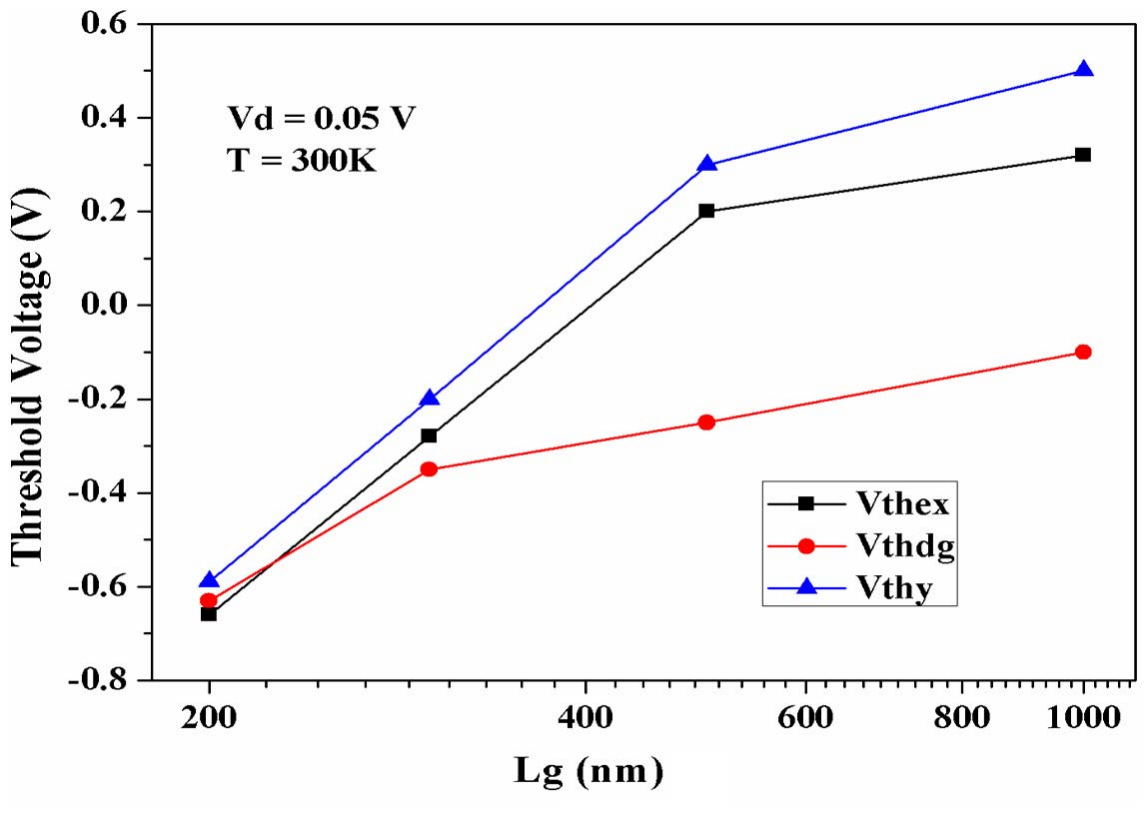
Threshold voltages (V_th_) vs L_g_ comparison extracted by different methods.

Several methods have been used for V_th_ extraction, which may show slight different in magnitude of V_th_ in accordance to these specific methods [16]. To investigate the impact of gate length variation on V_th_ value, threshold voltages were extracted by different methods. For the devices with different gate lengths, comparison of the threshold voltage values extracted by different methods, i.e. Y-function method (V_thy_), maximum of transconductance derivative (V_thdg_) and linear extrapolation of transfer characteristics (V_thex_) are demonstrated in Fig 5. It must be mentioned that it can be hard to find the precise values of the threshold voltage for V_thex_ and V_thy_ cases. For example, the linear extrapolation method is based on the linear change in the surface free charges and is under the influence of series resistance. Moreover, threshold voltage and flatband voltage can be hard to be identified from each other in linear extrapolation method as well as Y-function method [17], [18]. This can explain the different values of the threshold voltage for V_thex_ and V_thy_ in comparison with V_tdgm_ for larger gate length (Fig 5). Indeed, the V_thdg_ can offer more relevant information about threshold voltage, since it is extracted by the peak positions of the derivative of g_m_, and there is less effect of series resistance compared to the other methods.

To extract V_thy_, the correspondence Y-function is used,, which is defined as [19]: 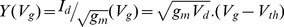
(1)


where V_d_ and V_g_ are the drain and gate voltage respectively. For different Lg with low drain voltage (V_d_ =  50 mV), V_thex_ and V_thdg_ values are extracted by using the maximum value of g_m_ and the position of first peak in the *dgm*/*dVg* plots respectively [16].

The overall comparison between all these different definitions of the V_th_ shows that roll-off of the threshold voltages due to the L_g_ scaling, which relates to SCE, regardless of what methods has been used. This comparison also demonstrates that the V_th_ shifts to the negative values for all extraction methods. The possible reason of this negative shift is the diffusion of dopant layer, at the interface of the InGaAs with the oxide layer. Recent works on X-ray photoelectron spectroscopy for doped InGaAs revealed that the monolayer of the dopant is present at the InGaAs interface even after the ALD oxide growth [20], [21]. The existence of border traps and defect states in ALD-Al_2_O_3_ dielectric interface with semiconductor is another source of diffusion [22].

The activation or annealing of III-V semiconductors at high temperature leads to more bulk defects, since it is involved with volatile V group. Therefore, the diffusion process is activated by high temperature PDA process and charges start to diffuse into the high-*K*/III-V interface. In our case, the PDA method was performed at 600°C which is considered as a high temperature PDA. Latest research [14] revealed that for PDA temperature more than certain value ( T > 400°C) the charge diffusion effect is increased. These charges can act as donor dopants in channel area and provide a depletion of the p-type channel [14] even at zero V_g_.

The large magnitude of the interface trap density (D_it_), is another cause for degradation of the charge control for the static and dynamic performance of the transistors. The interface trap density (D_it_) of ∼ 5.8×10^12^ eV^−1^cm^−2^ using CV measurements with HF-LF method and equation (2) [23], [24] has been calculated for the devices. 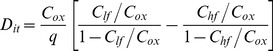
(2)


Here, C_ox_ is the fixed capacitance of the oxide layer and *q* is the fundamental charge, where C_lf_ and C_hf_ are the measured low and high frequency capacitance, respectively.

These causes can explain the negative shift in V_th_ observed for the gate first self-aligned In_0.53_Ga_0.47_As device (present work or for previous works [12], [25]). Moreover, extra negative charge can be imposed into the channel during the ion implantation process. The negative shift in V_th_ is not favourable for device performance, since it increases the *off*-state current, and it needs to be controlled by lower thermal budget [14]. Similar results have been reported for related works in In_0.53_Ga_0.47_As devices [7], [25].

**Figure 6:**
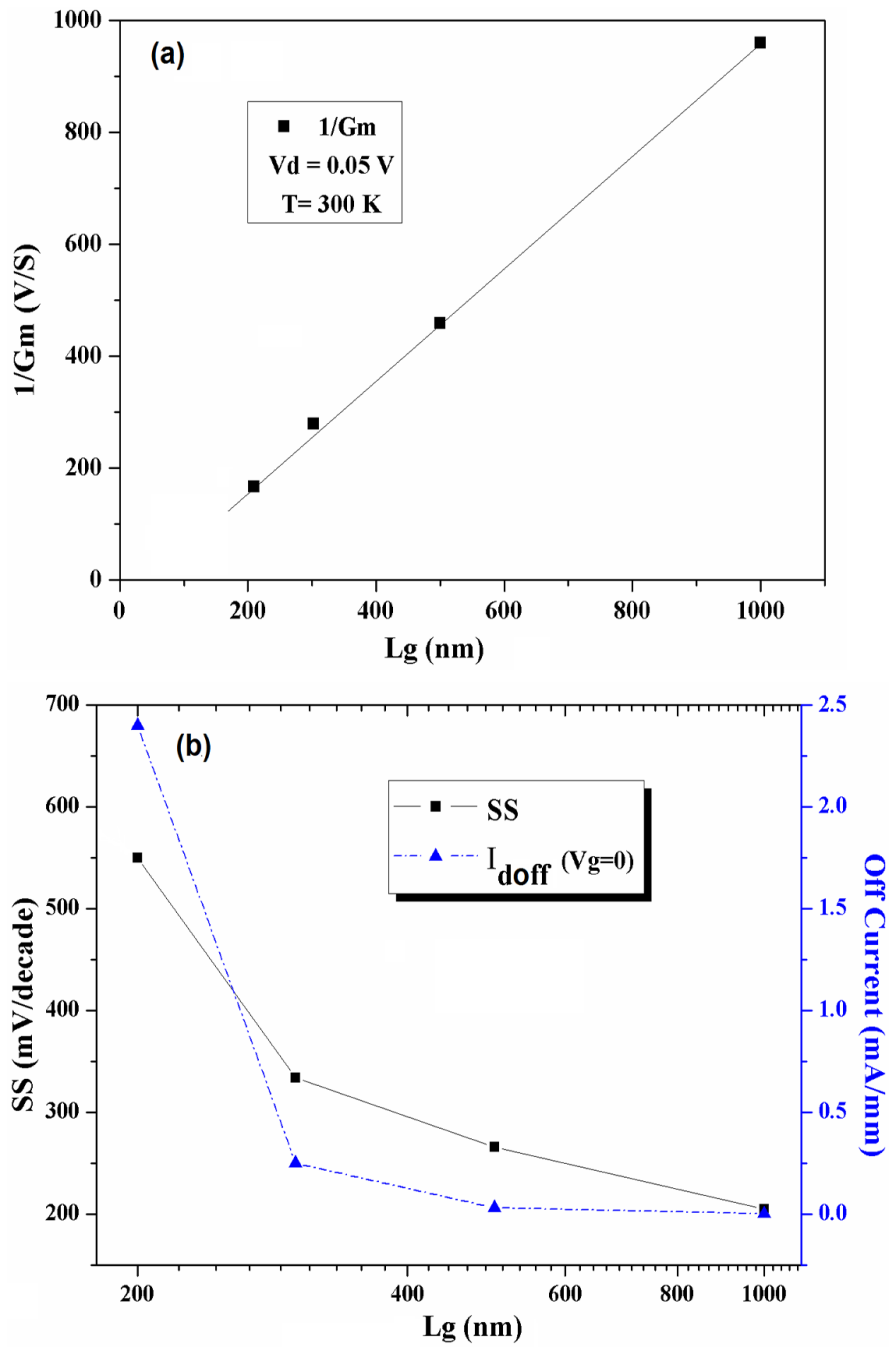
Variation of 1/G_m_ with gate length for ΔL extraction (a), Subthreshold swing and I_doff_ (b) for self-aligned In_0.53_Ga _0.47_As MOSFETs.

By using the Y-function method [19], transconductanse parameter (G_m_) for the low field effect mobility (orµ_o_, as an independent parameter with the gate length or width variation) can be extracted. For a fixed gate width, the plot of 1/G_m_ as a function of the L_g_ provides a straight line (Fig 6a), whose intercept with the gate length axis gives the gate to source/drain overlap length (ΔL).

The extracted value of ΔL from the plot shown in Fig 6a is 51 nm. There is a critical value for ΔL length above which the device performance and characteristics are suffered due to the scaling of transistors. On the other hand, there is an interaction of the overlap length with lateral doping abruptness which can affect the device performance, especially for scaled transistors [26]. Accordingly, knowing the value of ΔL is important for the device optimization, e.g. to find proper size of the overlap spacer for a device [27]. Conventionally, there is a minimum value for ΔL (∼20 nm) for 0.25 µm process, to avoid I_d_ degradation [28],http://www.jr.ietejournals.org/article.asp?issn=0377-2063year=2012volume=58issue=2spage=130epage=137aulast=Harish - ref12 but in recent works for the sub-100 nm regime, the smallest overlap length is strongly recommended.

The subthreshold swing (SS) and drain *off* state current (*I_doff_* at V_g_  =  0) variation for different gate lengths of the self-aligned In_0.53_Ga_0.47_As MOSFET device are shown in Fig 6b. Both results were derived from the transfer characteristics. The degradation in SS by decreasing the channel length can be seen. It probably is originated from the SCE, which also indicates that a higher rate of lateral diffusion has been introduced into the channel. As it can be seen in Fig 6b, variation of SS and *I_doff_* with the gate length has the same trend. In fact, higher SS value gives rise to a higher *off*-state current. This probably is more related to the large leakage current from drain junction than the intrinsic restriction regarding to the narrow band gap in InGaAs channel, which can be suppressed by more precise junction engineering. As it is shown in Fig 6b, the value of *I_doff_* is increased by reducing the gate length down to 200 nm, which reveals degradation in *off*-state performances of the device.

**Figure 7:**
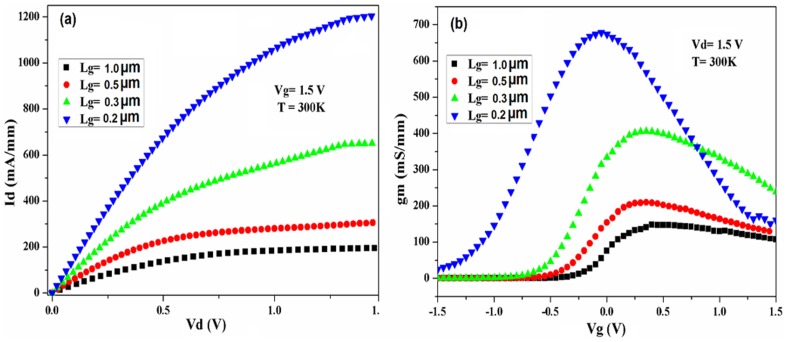
I_d_-V_d_ characteristics (a) and Transconductance vs V_g_ variation (b) of the devices for different L_g_.

The DC output characteristic comparison of the self-aligned In_0.53_Ga _0.47_As MOSFET for different gate lengths (1µm, 0.5 µm, 0.3 µm, 0.2 µm), at strong inversion layer (Vg  =  1.5V), are shown in Fig 7a. The drain current shows remarkable improvement due to the length reduction and highest current value extracted for shortest channel in the range of measurement, as 1.13 A/mm. This value is higher compared to the latest reported cases for drain current in self-aligned In0.53Ga 0.47As with the same gate size [29], [30]. Similar enhancement in intrinsic g_m_ is also observed in the devices with smaller L_g_. For g_m_, the highest value of 678 mS/mm was extracted for the device with L_g_ =  200 nm. Shorter L_g_ provides less resistance and lower surface-roughness scattering which leads to a higher transconductance and mobility for shorter L_g_. However, reducing the L_g_ resulted in presence of the SCE for the L_g_ smaller than 0.3µm, demonstrated in the form of increased I_d_ (not saturated) for the higher drain voltage. This behaviour including with degradation in *off*-state performances of device can be a sign of poor scalability of the device. For more improvement of the device performance, the optimization in fabrication process and development of low temperature activation technique are necessary, e.g using spacing wall between the gate and contacts, equivalent-oxide-thickness (EOT) reduction or spike rapid thermal annealing (RTA).

**Figure 8:**
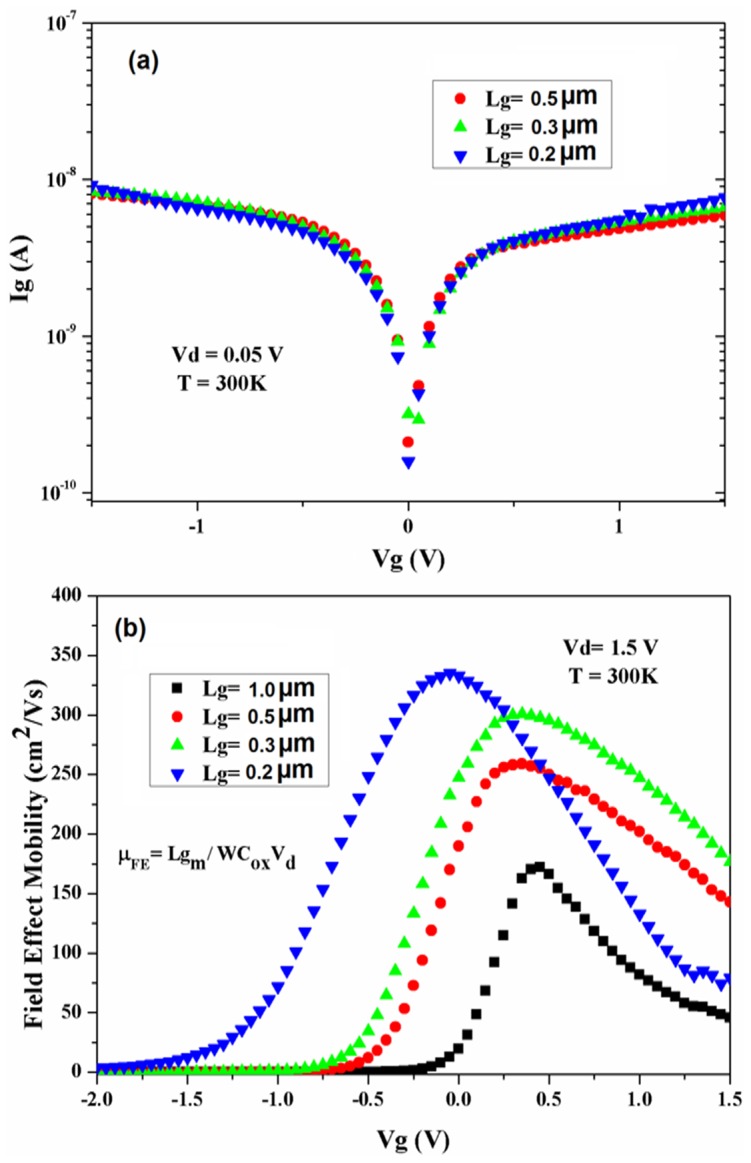
Gate current (a) and field effect mobility (b) versus V_g_ for different L_g_ for self-aligned In_0.53_Ga _0.47_As MOSFETs.

The effect of scaling the L_g_ on sub-threshold leakage current is shown in Fig 8a, where the Ig-Vg graph is shown for different L_g_ (V_d_  =  0.05V). Due to the gate voltage variation, the depletion (for negative gate voltage) and accumulation (for positive gate voltage) are recognisable in I_g_-V_g_ behaviour. As it can be seen, the level of sub-threshold leakage current (I_g_) for all devices with the different channel lengths are in order of 10^−8^ A which is acceptable range compare to the drain current. The value of I_g_ is low and has no change with the gate length variation showing a reliable performance of the high-*k* oxide layer.

Field-effect mobility (µ_FE_) behaviour versus gate voltage for devices with different gate lengths, extracted from the g_m_ analysis and equation (3) [31], is shown in Fig 8b. 
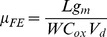
(3)


Here, L is the gate length, W is the channel width and C_ox_ is the oxide capacitance. Mobility of the devices were increased by scaling the L_g_ and theµ_FE_ peak of 364 cm^2^/Vs, was achieved for the length of Lg =  200 nm at V_d_  =  2 V and V_g_  = 1.5 V. The observedµ_FE_ enhancement for the device is related to the reduction of surface-roughness scattering by decreasing the L_g_. It also could be related to existence of less defects for shorter channel lengths, which also has important role for measured I_d_ enhancement (Fig7a) [32]. As it is expected, due to the higher intrinsic carrier mobility, theµ_FE_ value of the In_0.53_Ga _0.47_As MOSFET at a strong inversion region is higher than the GaAs MOSFET [33]. It is worth noting that the value of theµ_FE_ is generally less than the effective mobility which is normally extracted by split-CV method.

**Figure 9:**
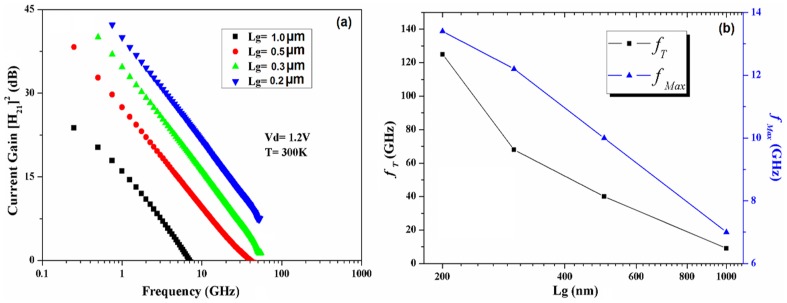
Extrinsic current gain H21 versus frequency (a), *f_T_* and *f_Max_* values (b) for different L_g_ of In_0.53_Ga _0.47_As MOSFETs.



The RF measurements and the S-parameters of the self-aligned n-type In_0.53_Ga _0.47_As MOSFET for different gate lengths were measured using a vector network analyser at room temperature. Fig 9a shows the extrinsic current gain ([H_21_]^2^) versus frequency for devices. By extrapolation of the extrinsic current gain (Fig 9a) and unilateral Masons’s gain (U_g_) from S-parameter measurements, the cut-off frequency (*f_T_*) and maximum oscillation frequency (*f_Max_*) were extracted, respectively. The gate length variation of the extracted *f_T_* and *f_Max_* for the devices are illustrated in Fig 9b. The results confirm the trend of increasing *f_T_* when the gate length decreases and the same trend were observed for *f_Max_*. The highest magnitude of 125 GHz was extracted for the device with L_g_  =  200 nm. The value of the *f_T_* is comparable with similar devices with 100 nm gate length [34]. In fact, the cut-off frequency (*f_T_*) increases by scaling MOSFETs [35], but the maximum oscillation frequency (*f_Max_*) is strongly depends on parasitic components of MOSFETs (like gate-drain and drain-source capacitances or gate resistance) [36]. The value of *f_Max_* can be approximately expressed as follows [37]:

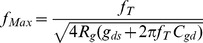
(4)where C_gd_ is the drain-to-gate capacitance, R_g_ is the effective gate resistance. It is noticeable from (4) that the terms appeared in the denominator, indicating the importance of these parameters

However, as it can be seen in Fig 9b, the value of *f_Max_* is low in comparison to the reported similar devices. It is probably related to the high value of the source/drain conductance (g_ds_) in our work, since the value of *f_Max_* has inverse proportionality with g_ds_. Another reason for having low value for *f_Max_* can be related to the chosen gate material, since the Ta metal gate provides high gate resistance, which lowers the value of *f_Max_*.

### Comparison with previous works

Research in the III-V industry began in the 60s specifically in 1967 with the oxides such as SiO_2_ and alumina, but the lack of knowledge on deposition techniques made unsuitable oxides. The development of ALD technology for depositing oxide layers is the key point for providing proper oxide thickness with proper materials. In order to give an overall comparison, the results presented here are compared with some of the previously published works as summarized in Table 1, in terms of I_d_, g_m_, gate length and gate dielectric thickness. Notice that only some of the enhancement-mode devices with inversion-channel are mentioned here, whose ALD preparation procedures were almost similar compared to the present work. There are other kinds of enhancement-mode III–V MOSFETs whose results are not mentioned here due to the limitation of space or different principles of device operation. Recently, InGaAs inversion- channel devices with even better performance and shorter gate length have been demonstrated, using InGaAs channels with higher In content (e.g. In_0.75_Ga_0.25_As).

**Table 1:** Brief summary of the previously published InGaAs inversion-channel MOSFETs.

Group	Channel/Material	Dielectric/Deposition/Oxide thickness	Characteristics
***Bell *** [***38***]	*Inversion/GaAs*	*UHV E-beam GGO,38nm*	*Lg = 1 µm; I_dmax_ = 30 µA/µm,* *g_m_ = 4 µS/µm*
***Purdue *** [***8***]	*Inversion,* *In0.65Ga0.35As*	*ALD-Al2O3, 10 nm*	*Lg = 0.4 µm; I_dmax_ = 1050 µA/µm;* *g_m_ = 350 µS/µm*
***IBM *** [***39***]	*Buried,* *In0.7Ga0.3As*	*ALD-Al2O3, 5.5 nm*	*Lg = 0.09 µm;I_dmax_ = 390 µA/µm;* *g_m_ = 610 µS/µm*
***Taiwan *** [***40***]	*Inversion,* *In0.53Ga0.47As*	*ALD-Al2O3, 6 nm*	*Lg = 0.6 µm; I_dmax_ = 678 µA/µm; g_m_ = 354 µS/µm*
***Taiwan *** [***13***]	*Inversion* *In0.75Ga0.25As*	*UHV E-beam evaporated Al2O3/GGO; = 2 nm/13nm*	*Lg = 1.2 µm; I_dmax_ = 960 µA/µm;* *g_m_ = 410 µS/µm*
***Lund University *** [***41***]	*Inversion,* *In0.53Ga0.47As*	*MOCVD-ALD -Al2O3- HfO2 = 0.5nm/6.5nm*	*Lg = 0.055 µm; I_dmax_ = 2000 µA/µm;* *g_m_ = 1900 µS/µm*
***Purdue *** [***42***]	*Inversion,* *In0.7Ga0.3As*	*ALD-Al2O3, 2.5 nm*	*Lg = 0.16 µm; I_dmax_ = 925 µA/µm;* *g_m_ = 1100 µS/µm*
***Tokyo *** [***43***]	*Inversion,* *In0.53Ga0.47As*	*ALD-Al2O3, 10 nm*	*Lg = 0.05 µm;I_dmax_ = 2400 µA/µm; g_m_ = 1170 µS/µm*
***Present Work***	*Inversion,* *In0.53Ga0.47As*	*ALD-Al2O3, 8 nm*	*Lg = 0.2µm; I_dmax_ = 1350µA/µm;* *g_m_ = 678µS/µm*

## Conclusions

Self-aligned n-type In_0.53_Ga _0.47_As with different gate length down to 200 nm were fabricated and characterized. The impact of gate length variation on device parameters such as threshold voltage, high and low voltage transconductance, subthreshold swing and *off* current are investigated at room temperature. Scaling the gate length revealed good enhancement in all investigated parameters but the negative shift in threshold voltage was observed for shorter gate lengths. The electron mobility value is lower and SS is higher as compared to silicon. The lower mobility is probably related to the higher D_it_ (10^12^) compared to silicon’s D_it_ (10^11^), which is due to gate first process and probably passivation method. The presence of the sidewall to control the lateral diffusion could be the solution for a lower SS and reducing the short channel effect. However, the device still manages to deliver a high drain current and transconductance compare to silicon. The results of RF measurement for cut-off and maximum oscillation frequency for devices with different gate lengths are compared. The results shows that a possibility is wide open for low power performance; however the high frequency performance needs to be improved. Thermal budget need to be adjusted for lowering the diffusion effect.
